# Depression among married female adolescents in Bangladesh: the toll of marriage, pregnancy, and violence

**DOI:** 10.7189/jogh.15.04033

**Published:** 2025-07-11

**Authors:** Md Mahabubur Rahman, Md Tazvir Amin, M Moinuddin Haider, Shusmita Hossain Khan, Sumaiya Nusrat, Quamrun Nahar, Mizanur Rahman, Kanta Jamil

**Affiliations:** 1 Health Systems and Population Studies Division (HSPSD), International Centre for Diarrhoeal Disease Research, Bangladesh (icddr,b), Dhaka, Bangladesh; 2 Data for Impact (D4I), Carolina Population Center, University of North Carolina at Chapel Hill, Chapel Hill, North Carolina, USA; 3 Maternal and Child Health Division (MCHD), International Centre for Diarrhoeal Disease Research, Bangladesh (icddr,b), Dhaka, Bangladesh; 4 Consultancy, Fitzroy North, Victoria, Australia

## Abstract

**Background:**

Major depressive disorder (MDD) is a critical psychiatric condition that is spreading faster among adolescents than adults around the globe. In low and middle-income countries, married female adolescents are more vulnerable to MDD than unmarried female adolescents. We aimed to estimate the prevalence of MDD among currently married female adolescents (CMFAs) in Bangladesh and determine its association with sociodemographic factors, factors related to marriage and childbearing, and social safety.

**Methods:**

We used data from the Bangladesh Adolescent Health and Well-being Survey (BAHWS) 2019–20, the first national-level survey conducted on adolescents ages 15–19 in Bangladesh. The BAHWS 2019–20 included a total of 2408 CMFAs aged 15–19 years and collected depression data using the modified Indian Bangla version of the Patient Health Questionnaire-9 (PHQ-9), which is specific to adolescents. We used multivariable logistic regression and Poisson regression with robust variance to examine the factors associated with MDD.

**Results:**

The estimated prevalence of MDD among CMFAs aged 15–19 years is 14.6%. The odds of MDD did not vary by age, education, wealth, residence type, or region. The CMFAs who felt connected with their husband and mother-in-law were 31% (adjusted odds ratio (aOR) = 0.69; 95% confidence interval (CI) = 0.53–0.91) and 34% (aOR = 0.66; 95% CI = 0.47–0.92) less likely to have MDD than the rest, respectively. The odds of MDD were almost 2-fold (aOR = 1.77; 95% CI = 1.24–2.52) among pregnant CMFAs than their non-pregnant counterparts. We found significantly higher odds of MDD among CMFAs who experienced physical violence from their husbands (aOR = 1.93; 95% CI = 1.31–2.85) or others (aOR = 2.07; 95% CI = 1.15–3.71), any form of sexual harassment (aOR = 2.25–95% CI = 1.49–3.42), and cyberbullying (aOR = 3.73; 95% CI = 2.01–6.93) than those who did not experience such adverse events.

**Conclusions:**

We observed a high prevalence of MDD among CMFAs, without clustering in any specific sociodemographic group. It is associated with not feeling connected with husband and mother-in-law, having a health condition (pregnancy), and experiencing physical, sexual, and cyber abuse. Findings suggest integrating mental health programs in maternity care and preventing violence against women may help prevent and manage MDD.

Major depressive disorder (MDD), commonly known as depression, is a critical mental disorder and a key measure of psychological well-being [1]. It reflects a person’s mental health and is indicative of underlying mental disorders and quality of life [2,3]. In 2019, depressive disorders accounted for 37% of 125 million disability-adjusted life years [4]. In recent years, depression has been spreading faster among adolescents than in adults and has become one of the top five illnesses in this subgroup in terms of prevalence [5,6].

It is worth noting that adolescents undergo a psycho-physical transition into becoming adults [7–11]. Their families and society also often impose various rules on them without considering their compliance, further affecting their psychological well-being [12–14]. Due to the vast societal differences, the burden and reasons for adverse psychological conditions like depression between low- and lower-middle-income countries (LLMICs) and high-income countries may vary. A national-level survey from Bangladesh (an LLMIC) showed that, compared to unmarried female adolescents, married female adolescents (MFAs) were more likely to have depression [6]. Depression among MFAs has an adverse impact on maternal and newborn health outcomes because they have either started childbearing or may initiate childbearing soon [15–17]. This makes understanding the factors associated with depression among MFAs in LLMICs crucial.

The social circumstances in LLMICs allow girls less freedom than boys [18]. Child marriage is one of the maladaptive practices of LLMICs affecting adolescent girls’ physical and psychological well-being. After marriage, they enter a complex social matrix of gender role expectations (including childbearing) which are often incompatible with their age and psychological state [19]. In addition to marriage and childbearing, disrupted social dignity can be a significant source of depression. Social dignity is rooted in the sense of an individual's worth and respect within their social environment; as such, it can be severely affected by their experiencing some form of violence. Hence, in this study, we conceptualised the social contexts for depression among MFAs into four broad domains, *i.e.* factors related marriage, childbearing, social safety, and sociodemographics. Below we briefly discuss the possible associations of these domains with depression.

## Factors related to marriage

Early marriage can have a detrimental influence on an adolescent girl’s mind. This may begin with the act of marriage, as many adolescent girls in LLMICs are forced to marry without their consent, forfeiting opportunities for schooling, leisure activities with peers, parental nurturing, and typical sibling interactions [6,20–22]. After marriage, the new bride usually relocates to her in-laws’ home, where she has to adjust to entirely new surroundings and responsibilities [6]. In parallel with the complexities of adolescence, during which they may find it difficult to comprehend or accept even well-intentioned guidance, the newlywed bride faces the burden of conforming to the new customs and expectations enforced by her husband and in-laws. Failure to meet the husband’s and in-laws’ expectations often result in verbal harassment and may lead to physical violence from the husband’s side [21]. These factors may severely disrupt the bride’s normal functioning, possibly resulting in an unstable psychological condition like depression.

In contrast, a harmonious relationship with the husband fosters an atmosphere where the bride may share her problems, find solutions, share her workload, and enjoy quality time with her husband, resulting in a sound psychological state [23,24]. A similar harmonious relationship with the in-laws may help the adolescent bride cope with the new challenges that arise with marriage [25]. For example, a mother-in-law can help the bride understand the in-laws’ norms and customs, arrange her needs, protect her from verbal or physical violence, and help the couple build a stable relationship, which may further result in lower levels of MDD.

## Childbearing

In LLMICs, one type of psychological pressure that in-laws often place on a bride is to conceive soon after marriage to prove her fertility [26,27]. This pressure is also rooted in the societal context, whereby delaying the first birth after marriage can cause rumors of infertility and disgrace the in-laws' family [28]. Thus, not conceiving soon after marrying may lead to familial and societal pressure being placed on MFAs, which in turn may be a cause of depression. Conversely, motherhood during adolescence further diminishes autonomy and amplifies pre-existing obligations, ultimately affecting their mental well-being. Pregnant adolescents also remain at risk of MDD during pregnancy due to hormonal shifts, psychological stressors, and pregnancy complications [29].

## Social safety

Social safety (*i.e.* safety from assault, chaos, and other form of violence) and an individual’s self-esteem are the second and fourth needs in Maslow’s hierarchical theory of human motivation, respectively [30]. Their infringement may affect psychological well-being [30]. Disrupted social dignity may further lead to depression, particularly in contexts where external forces undermine social identity and belonging like victimisation from any form of violence. Physical violence and harassment are examples of such external forces that lead to low self-esteem, trigger vulnerabilities, and develop beliefs about their fundamental worthlessness which may lead to MDD [31–33].

## Sociodemographic factors

Individual-level coping mechanisms for dealing with emotional fluctuations are one of the key drivers of experiencing depressive symptoms. Sociodemographic factors like age, education, socioeconomic status, residence type, *etc.*, may shape these coping mechanisms.

There is a paucity of research on depression among MFAs of LLMICs. One study from Indonesia highlighted the burden of depression among adolescent girls who had married early using a sample of only 76 adolescents [34]. Another study from India explored depression only among ever-pregnant adolescents during the COVID-19 pandemic [35]. Two studies from Bangladesh assessed depression among MFAs: one attempted to measure depression during the COVID-19 pandemic in a sub-district [36], while another focussed on the association of current pregnancy and spousal connectedness with depression [37]. None of these studies comprehensively investigated depression while focussing on factors related to marriage (*i.e.* wantedness of marriage, duration of marriage, connectedness with mother-in-law), childbearing (*i.e.* pregnancy and teen motherhood), and violence (*i.e.* physical violence, sexual harassment, cyberbullying).

Therefore, we aimed to test the following hypotheses in the LLMIC context:

1. A harmonious relationship with the husband and mother-in-law is associated with a lower risk of MDD among MFAs.
2. Childbearing and pregnancy are associated with a higher risk of MDD among MFAs.
3. Experiencing violence is associated with a higher risk of MDD among MFAs.

## METHODS

### The case of Bangladesh

Bangladesh, a South Asian LLMIC, has the eighth largest population in the world, with around 16.5 million female adolescents [38]. It is one of the top 10 countries with the highest observed child and adolescent marriage in the world (50.1%) [19,39]. Nearly half (an estimated 43%) of adolescent brides prefer not to marry during adolescence, but are forced to do so by the bride’s parents or relatives [21,22]. Further, victimisation is common in Bangladesh; physical violence perpetrated by husbands has historically been a substantial problem, while sexual harassment and cyberbullying have become emerging concerns [21,40–42]. However, studies on MDD among Bangladeshi married female adolescents is limited. We thus opted to test the above-listed hypotheses in Bangladesh, as an example of an LLMIC.

### Design, data, and participants

This study used publicly available secondary data from the cross-sectional Bangladesh Adolescent Health and Well-being Survey (BAHWS) 2019–20, the first national-level survey conducted on male-female and married-unmarried adolescents ages 15–19 years in Bangladesh [6]. The BAHWS used two-stage stratified sampling representative at the national, rural-urban, and regional (based on administrative divisions) levels. Rural and urban areas of eight administrative divisions formed sixteen strata. In the first stage, 728 clusters (primary sampling unit (PSU)) were drawn from the strata using the ‘probability proportional to size’ sampling method. In the second stage, 100 households were selected with equal probability from each cluster. All adolescents in these households were approached to participate in the survey. With a 97% response rate, BAHWS collected data from 4926 ever-married female adolescents.

**Figure 1:**
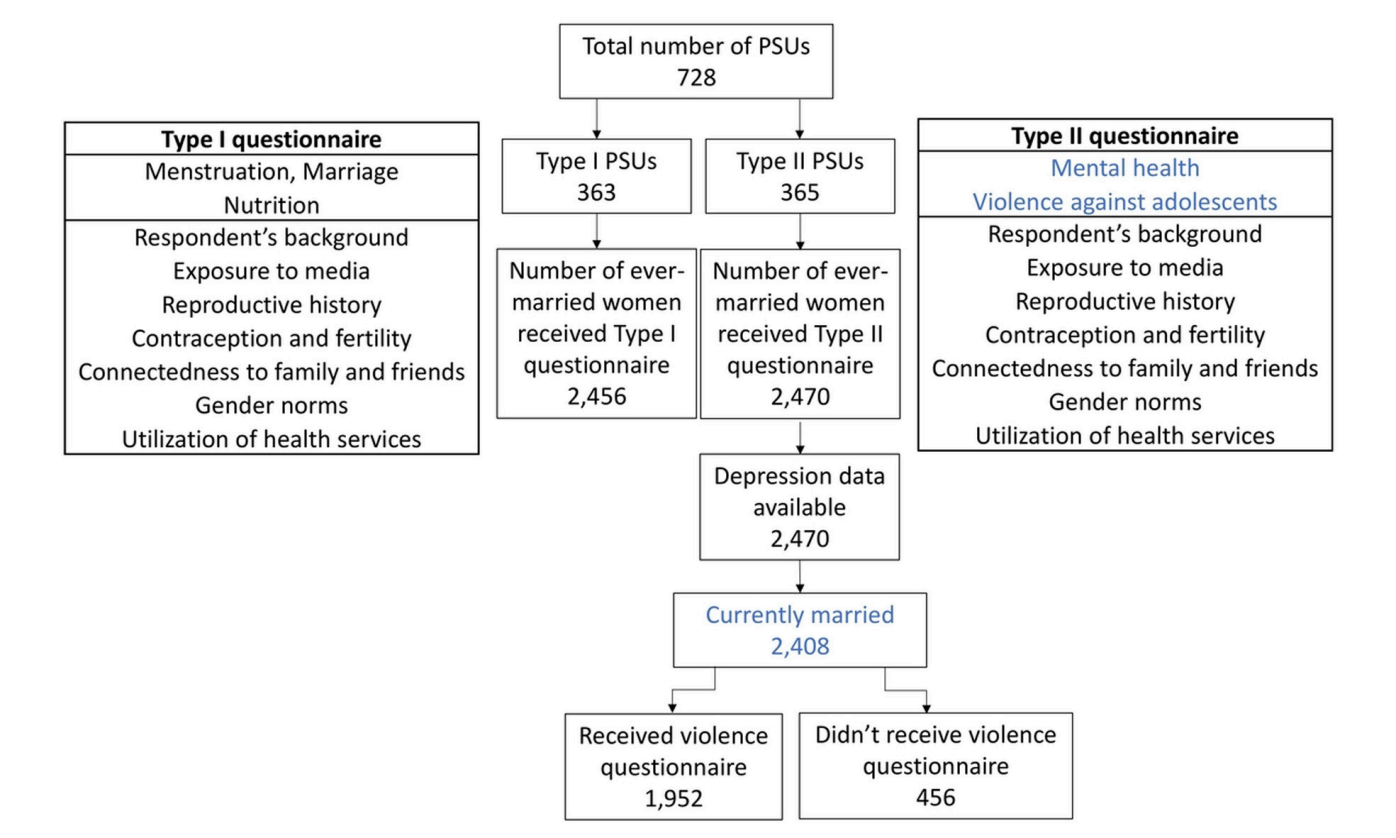
Data flow and questionnaire types.

Estimates of all the indicators do not require the same sample size. Thus, to reduce the survey cost of this national survey, while maintaining a sufficient sample size for all the indicators, BAHWS 2019-20 administered two modules of the questionnaire (Type I and Type II questionnaire) in two groups of PSUs. For this, PSUs were randomly divided into two groups (Type I and Type II PSUs) of equal number of PSUs within the combination of regional and rural/urban domains (**Figure 1**), which prevented the introduction of selection bias among respondents from either type of PSU. Type I and II questionnaires have a common module and a unique module. The unique module of the Type II questionnaire included mental health and violence questionnaires.

All adolescents from Type II PSUs received the mental health section. This included 2470 (unweighted) ever-married females, of whom 2408 were currently married (referred to as currently married female adolescents (CMFAs) here) and were thus included in our analytical sample. Further, 1952 of the study participants received questions on violence (which were considered sensitive questions), which followed the World Health Organization (WHO) ethical and safety recommendations for research on domestic violence [43]. The households of Type II PSUs with only one eligible adolescent received the violence questionnaire, while those with more than one eligible adolescent did not, as other eligible adolescents in the household may have become aware of the types of questions that the selected adolescent was asked. Maintaining privacy in this way was crucial, as any household member discovering that the adolescent had reported the violence to an interviewer could put the adolescent at risk for further abuse.

### Outcome measure

#### Tool development

The BAHWS 2019–20 used an Indian-Bengali version of the Patient Health Questionnaire-9 (PHQ-9), available at the PHQ Screeners website [44], and the PHQ-A, a modified version of PHQ-9 that is specific to adolescents [45], to draft a PHQ-9 screener for Bangladeshi adolescents. The reason for adopting the validated Indian-Bengali version was the language and cultural similarity between Bangladesh and the West Bengal state (adjacent to Bangladesh) of India. The newly developed draft of the PHQ-9 screener underwent rigorous pre-testing among the adolescent population (30 individuals in rural and 30 in urban areas) and was reviewed by subject matter experts before the final administration [6].

#### Outcome variable construction

The PHQ-9 consists of nine questions on a four-point Likert-type scale [44]. The BAHWS 2019–20 asked respondents about the presence of these nine items in the last two weeks of the survey. There were four possible choices for each item (‘Not at all’, ‘Some days’, ‘Majority of the days’, and ‘Nearly every day’), with scores ranging from 0 to 3 for each item. Thus, the total severity scores for a respondent ranged from 0–27 points.

The main outcome of interest was the presence of MDD, whereby respondents with PHQ-9 scores greater than or equal to 10 were considered to have MDD, while those who scored less than 10 were considered not to have MDD.

#### Covariates

From sociodemographic factors, we included age (15–17 or 18–19 years), educational attainment (primary completed, secondary incomplete, secondary completed or higher), household wealth quintile (lower two quintiles, middle quintile, upper two quintiles), type of residence (urban, rural), and region (western, central, eastern). We constructed the household wealth quintile using principal component analysis of durable assets and household construction materials.

The domain of marriage included five factors: preferred to marry then (right time or earlier, later), marriage duration in years (0–1, 2–3, ≥4), currently living with husband (yes, no), connectedness with husband (connected, not connected), and connectedness with mother-in-law (connected, not connected, lives elsewhere). The domain of childbearing included current pregnancy status (currently not pregnant, currently pregnant, unsure) and started childbearing (yes, no). The domain of social safety comprised two factors: experience of physical violence (no, only from husband, only from others, from husband and others, unknown) and experience of any sexual harassment or cyberbullying (no, only sexual harassment, only cyberbullying, both, unknown). We categorised the respondents who did not receive the violence questionnaire as ‘unknown’ in the factors of experience of physical violence, sexual harassment, and cyberbullying (**Table 1**).

**Table 1:** Variable description

	Description
**Sociodemographic factors**	
Age in years	
*15–17*	Current age is between 15–17 yearsears
*18–19*	Current age is between 18–19 years
Educational attainment	
*Primary*	Completed 1–5 years of schooling (only 58 CMFAs never attended school; they are also included here).
*Secondary incomplete*	Completed 6–9 years of schooling
*Secondary completed or higher*	Completed 10 years of schooling
Household wealth quintile	
*Lower two quintiles*	Belonged to first or second wealth quintile group
*Middle*	Belonged to third wealth quintile group
*Upper two quintiles*	Belonged to fourth or fifth wealth quintile group
Type of residence	
*Urban*	Lives in urban areas
*Rural*	Lives in rural areas
Region	
*Western*	Lives in Rangpur, or Rajshahi, or Khulna divisions
*Central*	Lives in Mymensingh, or Dhaka, or Barishal divisions
*Eastern*	Lives in Sylhet, or Chattogram division
**Factors around marriage**	
Preferred to marry then	
*Right time or earlier*	Preferred to marry then or earlier
*Later*	Preferred to marry later
Marriage duration in years	
*0–1*	Marriage duration is at most one year
*2–3*	Marriage duration is 2–3 years
*4+*	Marriage duration is at least 4 years
Currently living with husband	
*Lives with husband*	Currently living with husband
*Husband lives elsewhere*	Currently husband is living somewhere else
Connectedness with husband	
*Connected*	If a CMFA enjoys spending time with her husband and talking about very personal things with him most of the time
*Not connected*	Otherwise
Connectedness with mother-in-law	
*Connected*	If a CMFA enjoys spending time with mother-in-law most of the time
*Not connected*	Otherwise
*Lives elsewhere*	Mother-in-law lives elsewhere
**Childbearing**	
Pregnancy status	
*Currently not pregnant*	If a CMFA was not pregnant on survey day
*Currently pregnant*	If a CMFA was pregnant on survey day
*Unsure*	If a CMFA was unsure about pregnancy status on survey day
Started childbearing	
*No*	Did not give any birth yet
*Yes*	Gave at least one birth
**Social safety**	
Experience of physical violence in last one year	If someone slapped, pushed, pulled her hair, punched, threw something at her, hit by a stick or something heavy, kicked, dragged, beaten up, tried to choke or burnt her with something hot/acidic, threatened or attacked her with knife, gun or any other weapon in the last one year.
*No*	Did not experience physical violence
*Only from husband*	Experience physical violence from husband only
*Only from others*	Experience physical violence from other than husband
*From husband and from others*	Experience physical violence from husband and others
*Unknown*	Violence-related questions were not asked
Experience of any sexual harassment or cyberbullying in last one year	
*No*	Did not experience sexual or cyberbullying
*Only sexual harassment*	If a CMFA was starred at her in a vulgar manner that made her uncomfortable, encountered sly whistles or humming of suggestive songs, passed sexual comments or jokes, touched or grabbed or pinched that made her uncomfortable, forced to watch obscene photos or videos or flashed or mooned, or other sexual harassment in the last one year but did not experience cyberbullying
*Only cyberbullying*	If anyone harassed or bothered a CMFA, spread mean words about her, or shared pictures of her using a mobile phone or the internet in last one year but did not experience sexual harassment
*Experience both*	Experienced both sexual harassment and cyberbullying
*Unknown*	Violence-related questions were not asked

CMFA – currently married female adolescents

#### Statistical analyses

We performed univariate analyses to gain better insight into the data. We estimated the prevalence of MDD across different characteristics of CMFAs. We used a survey-weight-adjusted Rao-Scott chi-squared test of association to explore the association of covariates with MDD. Given that odds ratios and prevalence ratios are commonly used relative measures of association, we estimated adjusted odds ratios using multivariable logistic regression to identify factors independently associated with MDD.

In general, interpreting odds ratios as prevalence ratios is inadequate not only due to possible over-estimation, but also because confounding may not be controlled appropriately. Since confounding depends on the measure of effect, the approach to controlling for confounding when estimating odds ratios differs from that used for prevalence ratios [46,47]. Thus, we also performed a sensitivity analysis to assess whether the observed association is robust to model specification, wherein we used Poisson regression with robust variance. This approach provides a closer estimate of the Mantel-Haenszel prevalence ratio than the other regression models (logistic regression, Poisson regression with scale parameter adjusted by deviance, Poisson regression with scale parameter adjusted by χ^2^) for cross-sectional data with a binary outcome [48]. While log-binomial regression was another alternative, in the presence of multiple politomic covariates (as was the case with our data), estimates of Newton-Raphson method often do not converge to the maximum likelihood estimates [49].

We adjusted for the survey weight and survey design characteristics of the BAHWS 2019–20 to reduce the bias of the estimates and produce robust standard errors of the estimates. We carried out all statistical analyses in Stata, version 14.0 (StataCorp LLC, College Station, TX, USA).

## RESULTS

### Characteristics of currently married female adolescents

Around one-fifth (21.3%) of the respondents had primary education, and one-quarter (24.2%) completed secondary education (**Table 2**). Around two-thirds (63.1%) wanted to marry later than the age they married. About half (49.3%) had only been married for one year or less, and 82% lived with their husbands. Just under one-sixth (15.4%) of CMFAs were pregnant during survey time and nearly half (43.46%) had already given birth. About 12.2% reported being victims of intimate partner violence (IPV) in the last year. During the same period, 13.4% of CMFAs experienced sexual harassment, while 5.8% experienced cyberbullying.

**Table 2:** Characteristics of currently married female adolescents (n = 2408)

	Weighted number (%) of CMFAs	Number of CMFAs
**Sociodemographic factors**		
Age in years		
*15–17*	1046 (43.2)	1044
*18–19*	1376 (56.8)	1364
Educational attainment		
*Primary*	516 (21.3)	519
*Secondary incomplete*	1319 (54.5)	1305
*Secondary completed or higher*	587 (24.2)	584
Household wealth quintile		
*Lower two quintiles*	1000 (41.3)	984
*Middle*	553 (22.8)	565
*Upper two quintiles*	869 (35.9)	859
Type of residence		
*Urban*	570 (23.5)	537
*Rural*	1851 (76.4)	1871
Region		
*Western*	965 (39.8)	948
*Central*	953 (39.3)	845
*Eastern*	504 (20.8)	615
**Factors around marriage**		
Preferred to marry then		
*Right time or earlier*	893 (36.9)	892
*Later*	1529 (63.1)	1516
Marriage duration in years		
*0–1*	1193 (49.3)	1199
*2–3*	812 (33.5)	801
*4+*	416 (17.2)	408
Currently living with husband		
*Lives with husband*	1975 (81.5)	1951
*Husband lives elsewhere*	446 (18.4)	457
Connectedness with husband		
*Not connected*	1336 (55.2)	1320
*Connected*	1086 (44.8)	1088
Connectedness with mother-in-law		
*Not connected*	465 (19.2)	453
*Connected*	1374 (56.7)	1389
*Lives elsewhere*	583 (24.1)	566
**Childbearing**		
Pregnancy status		
*Currently not pregnant*	2022 (83.5)	2002
*Currently pregnant*	373 (15.4)	382
*Unsure*	27 (1.1)	24
Started childbearing		
*No*	1366 (56.4)	1362
*Yes*	1055 (43.6)	1046
**Social safety**		
Experience of physical violence in last one year		
*No*	1585 (65.4)	1586
*Only from husband*	262 (10.8)	251
*Only from others*	91 (3.8)	83
*From husband and from others*	34 (1.4)	32
*Unknown*	449 (18.5)	456
Experience of any sexual harassment or cyberbullying in last one year		
*No*	1578 (65.2)	1573
*Only sexual harassment*	254 (10.5)	250
*Only cyberbullying*	71 (2.9)	66
*Experience both*	70 (2.9)	63
*Unknown*	449 (18.5)	456
**Total**	2422 (100.0)	2408

CMFA – currently married female adolescents

### Prevalence of major depressive disorder

**Figure 2:**
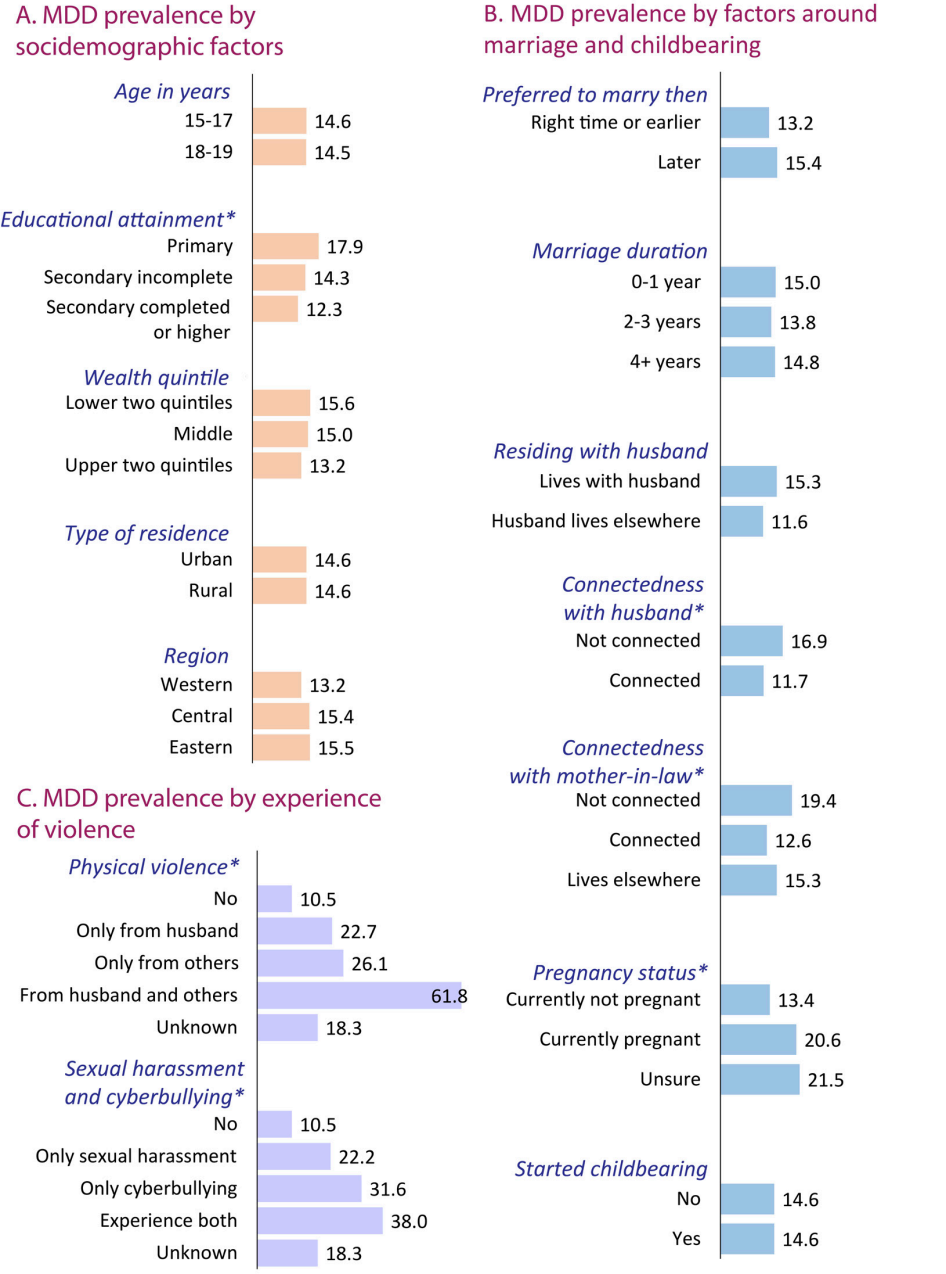
Prevalence of MDD among currently married female adolescents (n = 2408). *P-value of Rao-Scott χ^2^ test is less than 0.05. MDD – major depressive disorder.

Overall, the prevalence of MDD was 14.6%. The χ^2^ test of association suggested significant differences in MDD prevalence by educational attainment, connectedness with husband and mother-in-law, pregnancy status, and experience of physical, sexual, and cyber harassment (**Figure 2**).

The prevalence of MDD was 12% among CMFAs connected with their husband, compared to 17% among their counterparts. Connectedness with mother-in-law appears to be a great source of variation in MDD prevalence. Specifically, 19% of CMFAs who were not connected with their mother-in-law had MDD, compared to only 13% of those who were connected. The prevalence of MDD was higher among pregnant CMFAs (21%) than their counterparts (13%).

The prevalence of MDD among CMFAs who had not experienced physical violence was 11%, whereas it was more than double that among those who had. Specifically, the prevalence of MDD among those CMFAs who had encountered sexual harassment or cyberbullying was 22% and 32%, respectively, which was more than 2-fold than those who did not experience such events.

### Factors associated with major depressive disorder

The logistic regression and Poisson regression with robust variance yield the same direction of the association between the covariates and MDD. As the prevalence of MDD was 14.6%, indicating that the outcome event is neither highly rare nor highly common, the odds ratios remained close to the prevalence ratios, with a slight overestimation. This suggests that the observed association of the covariates with MDD was robust to model specification.

Adjusted odds ratios (aOR) and prevalence ratios suggest that none of the sociodemographic factors were associated with MDD (**Table 3**). Among the factors around marriage, connectedness with the husband and the mother-in-law were associated with lower odds of MDD. The CMFAs who were connected with their husbands and mother-in-law were 31% and 34% less likely to have MDD, respectively, than those who were not connected. The Poisson model suggested that the prevalence of MDD was 25% and 28% lower among CMFAs who were connected with their husbands and their mother-in-law, respectively, than among those who were not.

**Table 3:** Correlates of MDD: results from multiple logistic regression and Poisson regression with robust variance (n = 2408)

				

	aOR (95% CI)	*P*-value	aPR (95% CI)	*P*-value
**Sociodemographic factors**				
Age in years				
*15–17*	ref		ref	
*18–19*	1.15 (0.87–1.52)	0.316	1.10 (0.88–1.39)	0.405
Educational attainment				
*Primary*	ref		ref	
*Secondary incomplete*	0.83 (0.61–1.13)	0.240	0.85 (0.67–1.08)	0.196
*Secondary completed or higher*	0.77 (0.50–1.19)	0.240	0.81 (0.59–1.11)	0.188
Household wealth quintile				
*Lower two quintiles*	ref		ref	
*Middle*	0.99 (0.70–1.40)	0.960	1.00 (0.77–1.29)	0.999
*Upper two quintiles*	0.84 (0.61–1.16)	0.288	0.87 (0.67–1.12)	0.276
Type of residence				
*Urban*	ref		ref	
*Rural*	1.09 (0.80–1.48)	0.592	1.08 (0.82–1.42)	0.597
Region				
*Western*	ref		ref	
*Central*	1.25 (0.94–1.66)	0.131	1.18 (0.94–1.48)	0.149
*Eastern*	1.28 (0.92–1.78)	0.140	1.19 (0.89–1.59)	0.242
**Factors around marriage**				
Preferred to marry then				
*Right time or earlier*	ref		ref	
*Later*	1.09 (0.83–1.42)	0.531	1.09 (0.86–1.36)	0.480
Marriage duration in years				
*0–1*	ref		ref	
*2–3*	0.88 (0.57–1.35)	0.550	0.91 (0.67–1.24)	0.550
*4+*	0.90 (0.57–1.43)	0.655	0.93 (0.63–1.37)	0.714
Currently living with husband				
*Lives with husband*	ref		ref	
*Husband lives elsewhere*	0.76 (0.53–1.09)	0.132	0.81 (0.61–1.07)	0.141
Connectedness with husband				
*Not connected*	ref		ref	
*Connected*	0.69 (0.53–0.91)	0.007	0.75 (0.60–0.93)	0.008
Connectedness with mother-in-law				
*Not connected*	ref		ref	
*Connected*	0.66 (0.47–0.92)	0.016	0.72 (0.56–0.92)	0.010
*Lives elsewhere*	0.78 (0.52–1.16)	0.217	0.81 (0.60–1.09)	0.162
**Childbearing**				
Pregnancy status				
*Currently not pregnant*	ref		ref	
*Currently pregnant*	1.77 (1.24–2.52)	0.002	1.55 (1.18–2.02)	0.001
*Unsure*	1.15 (0.36–3.69)	0.814	1.08 (0.51–2.30)	0.843
Started childbearing				
*No*	ref		ref	
*Yes*	1.15 (0.77–1.71)	0.492	1.12 (0.82–1.53)	0.486
**Social safety**				
Experience of physical violence in last one year				
*No*	ref		ref	
*Only from husband*	1.93 (1.31–2.85)	0.001	1.70 (1.26–2.32)	
*Only from others*	2.07 (1.15–3.71)	0.015	1.81 (1.17–2.78)	0.405
*From husband and from others*	7.81 (3.68–16.60)	<0.001	3.27 (2.24–4.77)	
*Unknown*	2.33 (1.67–3.24)	<0.001	2.01 (1.53–2.65)	
Experience of any sexual harassment or cyberbullying in last one year				0.196
*No*	ref		ref	0.188
*Only sexual harassment*	2.25 (1.49–3.42)	<0.001	1.92 (1.41–2.62)	
*Only cyberbullying*	3.73 (2.01–6.93)	<0.001	2.70 (1.84–3.98)	
*Experience both*	3.82 (1.95–7.46)	<0.001	2.55 (1.70–3.83)	0.999

aOR – adjusted odds ratio, aPR – adjusted prevalence ratio

From both models, we noticed a significant MDD differential by pregnancy status. The odds and prevalence of MDD were respectively 1.77 and 1.55 times higher among pregnant CMFAs than non-pregnant.

Experiencing physical violence, sexual harassment, and cyberbullying was significantly associated with a greater likelihood of MDD. Both the odds and prevalence of MDD were almost 2-fold among CMFAs who experienced IPV *vs.* those who did not experience any physical violence. The odds of MDD further multiplied (aOR = 7.81; 95% CI = 3.68–16.60) when physical violence from other perpetrators was added to IPV. However, in this case, the prevalence ratio of 3.27 (95% CI = 2.24–4.77) was quite lower than the odds ratio. The CMFAs who experienced only sexual harassment were two times as likely to have MDD than those who did not. Importantly, the odds of MDD were almost four times for those who experienced cyberbullying. Similarly, for this case, the Poisson model suggests the prevalence ratio to be around three times higher.

## DISCUSSION

### Intimate partner violence and depression

Our findings suggest that two-thirds of the CMFAs in our sample wanted to marry later at the time of their marriage. However, this unwillingness was not associated with an adverse outcome like MDD. The only marriage-related factors associated with a lower chance of MDD were connectedness with the husband and mother-in-law. In contrast, IPV was associated with a higher chance of MDD, which is supported by earlier literature from both high- and low-income countries [50,51]. Encouragingly, the burden of IPV in Bangladesh is decreasing, albeit at a slow pace [21].

### Lack of connectedness with mother-in-law and depression

The relationship between a mother and her daughter-in-law is complex. The two are not connected by blood, but commonly live in the same needs-network. As they often reside in the same household, one can support each other’s needs, and a harmonious relationship between them can improve their daily lives. The mother-in-law usually has substantial access to the bride and groom and holds an influential position in the household. She can therefore protect the bride from the negative experiences. We found that, among CMFAs, feeling connected with the mother-in-law was significantly associated with a lower risk of MDD.

### Pregnancy and depression

Hormonal changes due to pregnancy increase the risk of onset or recurrence of depression, leading to a higher level of MDD among pregnant than non-pregnant women [52]. Supporting this, we also found a significantly higher prevalence of MDD among pregnant (20.6%) than non-pregnant CMFAs (13.4%). A meta-analysis using 33 studies from South Asian countries estimated the pooled prevalence of antenatal depression to be 24.3% (95% CI = 19.03–30.47), supporting our estimates of MDD prevalence (20.6%) for pregnant CMFAs [53].

### Physical, sexual and cyber abuse and depression

Our study is the first to show that physical violence, sexual harassment, and cyberbullying are significantly associated MDD among Bangladeshi CMFAs. In most cases, household members, neighbours, and friends were the perpetrators of physical violence [6]. Earlier literature also supports our findings on the higher level of MDD among sexually harassed and cyberbullied adolescents [54–58].

### Recommendations

#### Programmes to reduce IPV

According to the literature, IPV may occur when a wife commits any perceived mistake, tries to restrict her husband from doing something, or argues with in-laws, particularly her husband [21]. Therefore, empowering women through education and employment, and through gender sensitisation programmes targeted at men and male adolescents, can help decrease IPV in society. Utilising law enforcement and establishing a helpline for emergency response to IPV is also required.

#### Social transformation to improve relationship between daughter and mother-in-law

Our results show that one in every five CMFAs do not feel connected with their mother-in-law. Interventions addressing the social and cultural factors aimed at forming a harmonious relationship between the two are imperative. For example, cultural sensitivity training aimed at bridging the cultural and generational gap, alongside training on conflict resolution approaches, may help navigate disagreements and improve the relationship between the bride and the mother-in-law.

#### Integration of mental healthcare in maternity care

We estimated the prevalence of MDD among pregnant CMFAs to be 20.6%, which would translate to around 200 000 pregnant CMFAs suffering from MDD every year [59,60]. Prenatal depression is one of the strongest risk factors for postpartum depression [61,62]. Thus, introducing mental health services dedicated to pregnant women is of utmost importance. The high uptake of antenatal care (ANC) opens the possibility of screening and treating MDD among pregnant women. However, MDD screening is not yet included in ANC components, and there is no national guideline for treating a pregnant woman with MDD symptoms. Recent implementation research aimed at enhancing access to mental health services, conducted in six government facilities in Bangladesh, has prioritised pregnant women (among other groups) [63]. The ANC providers refer pregnant women to the well-being centre of the facility, where after initial screening by a health worker, psychologists counsel women with psychological symptoms remotely in the well-being centre and refer them to dedicated psychiatrists (if necessary) who also treat them remotely at a scheduled time in this well-being centre [63]. They showed that the well-being centre model is feasible and useful, but the main challenge is the scarcity of trained staff. Therefore, we suggest including MDD screening in ANC components and developing a national guideline for treating prenatal depression.

#### Enforcement of law and improving social norms to reduce violence against women

To combat gender-based violence against women, the Government of Bangladesh (GoB) has set up a 24-hour helpline (‘109’) and a mobile app (‘Joy’), alongside the established laws and punitive actions for sexual harassment and cybercrime [41,64]. We suggest that the next steps is to ensure complete enforcement of existing laws, which may help build a safe society with less MDD among CMFAs. Nurturing healthy social norms is also essential to prevent violence against women. For example, another child is frequently the perpetrator of the violence. Therefore, we propose the establishment of school-based education programmes promoting equitable gender roles and non-violent conflict resolution from an early age. Moreover, community-based programmes engaging men and boys aimed at promoting positive masculinity and condemning violence may be of help in this sense.

#### Contact points for introducing mental health aspects

An inclusive approach should be taken so that no married girl is left unaware of mental health rights and services. Hence, mental health awareness interventions should be accessible before marriage, after marriage, during pregnancy, and following childbirth. As girls’ participation in secondary-level education is increasing in Bangladesh, the inclusion of mental health education in textbooks focusing on mental health rights, coping mechanisms, and whom to discuss can be a possible way of informing adolescents. However, the health education contents of the national curriculum are mainly limited to reproductive health. After marriage, a married adolescent can be informed about mental health aspects by the family welfare assistants (FWA) who visit door to door to counsel couples on family planning. Even if FWAs do not get enough time to discuss mental health, at least they can deliver a booklet containing the necessary information. ANC and post-natal care (PNC) can be another two vital contact points for discussing mental health aspects with female adolescents.

#### Adequacy of government actions to improve mental health

Mental health has historically received little attention in Bangladesh due to the high burden of other physical health crises. Estimates suggest there to be just one psychiatrist for every 200 000 population, and that the average amount spent by the GoB on mental health is only USD 0.08 per person yearly [65]. However, the GoB has recently undertaken several initiatives to address the mental health needs of citizens. For example, Bangladesh is among the first few countries in the WHO South-East Asia Region to place mental health as one of its top 10 priority health conditions [65]. The GoB further enacted the Mental Health Act 2018 and approved the National Mental Health Strategic Plan 2020–30 for implementation [66]. To accomplish Sustainable Development Goal 3.4, the Ministry of Health and Family Welfare pledged in its fourth five-year Health Population and Nutrition Sector Program 2017–22 to make ‘mental health and psychosocial well-being’ a priority area within the existing healthcare system [66]. The GoB has also declared adolescent mental health as one of the four priority thematic areas of Adolescent Health Strategy 2017–30 [67]. Accordingly, the first strategic objective involves integrating the mental health agenda within primary healthcare services.

### Strengths and limitations

We used the cut-off point of ≥10 of the PHQ-9 scale, modified for adolescents, to measure MDD. The sensitivity and specificity for this cut-off are as high as 88% [6]. Due to our use of secondary data, we could not use the Edinburgh Postnatal Depression Scale or the Center for Epidemiologic Studies Depression Scale; however, we note that the PHQ-9 score cut-off shows high concordance (84% sensitivity and specificity) with the Structured Clinical Interview for DSM-IV [68]. Furthermore, we used a nationally representative sample, which allowed us to estimate the national-level prevalence of MDD among CMFAs. We performed the analyses using appropriate survey weights and survey design characteristics, which reduced bias from the estimates and yielded robust standard errors for the estimates.

Establishing any causal relationship of the factors associated with MDD was beyond the scope of the study. Due to our use of secondary data, we could not adjust for some potential confounders (such as previous mental health histories and other psychological stressors) that might have influenced both the timing of the marriage and the risk of MDD. In addition, due to data unavailability, we could not control for comorbidities that may lead to MDD. Though earlier literature showed a positive association between childbearing and MDD, we found no such evidence in our data [69,70]. In our analyses, the group ‘Did not start childbearing’ included five groups of CMFAs: those who wanted to have a child, but failed due to fertility problems; those who could conceive, felt peer pressure to do so, but did not want to have a child at the time of the survey; those who could conceive, felt peer pressure to do so, and wanted to have a child at the time of the survey; those who could conceive, did not feel peer pressure to do so, and did not want to have a child at the time of the survey; and those who could, did not feel peer pressure to do so, but wanted a child at the time of the survey,. The first two groups and the last one may have had potential reasons (infertility and peer conflict to conceive) for MDD. Conversely, those who started childbearing may have had depression due to additional burdens related to the child. It is possible that, if we were able to disaggregate the ‘Without-child’ group into the above-mentioned categories, we might have observed similar prevalence of MDD among CMFAs with or without a child.

## CONCLUSIONS

In this study, we estimated the prevalence of MDD among CMFAs aged 15–19 years in Bangladesh and identified its correlates of MDD. The findings highlight no significant differential in MDD prevalence by education, type of residence, and geographical region. We observed, however, that connectedness with the husband and mother-in-law were protective factors for MDD. We further found that every fifth pregnant CMFA is experiencing depressive symptoms, highlighting the necessity of integrating screening and treating depression at ANC corners. Furthermore, experiencing physical violence, sexual harassment, and cyberbullying were the key correlates of higher MDD. This emphasises the need for developing social transformation interventions promoting gender equity, positive masculinity, and non-violent conflict resolution targeted at men and boys, as well as ensuring the enforcement of laws to condemn violence.

## References

[1] Joseph S De Guzman R Relationship between psychological well-being and depression among selected adolescents. Indian J Posit Psychol. 2021;12:232–5.

[2] Singh A Misra N Loneliness, depression and sociability in old age. Ind Psychiatry J. 2009;18:51. 10.4103/0972-6748.57861 21234164 PMC3016701

[3] Viertiö S Kiviruusu O Piirtola M Kaprio J Korhonen T Marttunen M Factors contributing to psychological distress in the working population, with a special reference to gender difference. BMC Public Health. 2021;21:611. 10.1186/s12889-021-10560-y 33781240 PMC8006634

[4] GBD 2019 Mental Disorders Collaborators Global, regional, and national burden of 12 mental disorders in 204 countries and territories, 1990–2019: a systematic analysis for the Global Burden of Disease Study 2019. Lancet Psychiatry. 2022;9:137–50. 10.1016/S2215-0366(21)00395-3 35026139 PMC8776563

[5] Miller L Campo JV Depression in adolescents. N Engl J Med. 2021;385:445–9. 10.1056/NEJMra2033475 34320289

[6] National Institute of Population Research and Training (NIPORT), International Centre for Diarrhoeal Disease Research, Bangladesh. (icddr,b), Data for Impact. Bangladesh Adolescent Health and Wellbeing Survey 2019-20: Final Report. Dhaka, Bangladesh, Chapel Hill, NC, USA: NIPORT, icddr,b, Data for Impact; 2021. Available: https://www.data4impactproject.org/publications/bangladesh-adolescent-health-and-wellbeing-survey-2019-20-final-report/. Accessed: 5 May 2025.

[7] Kujawa A Burkhouse KL Vulnerability to depression in youth: Advances from affective neuroscience. Biol Psychiatry Cogn Neurosci Neuroimaging. 2017;2:28–37. 10.1016/j.bpsc.2016.09.006 28497126 PMC5421558

[8] Bergland C. Why Is the Teen Brain So Vulnerable?. Psychology today. 19 December 2013. https://www.psychologytoday.com/us/blog/the-athletes-way/201312/why-is-the-teen-brain-so-vulnerable. Accessed:5 May 2025.

[9] United Nations Children’s Fund. The state of the world's children 2011-adolescence: an age of opportunity. New York, NY, USA: United Nations Children's Fund (UNICEF); 2011. Available: https://www.unicef.org/media/84876/file/SOWC-2011.pdf. Accessed: 5 May 2025.

[10] Kaltiala-Heino R Marttunen M Rantanen P Rimpelä M Early puberty is associated with mental health problems in middle adolescence. Soc Sci Med. 2003;57:1055–64. 10.1016/S0277-9536(02)00480-X 12878105

[11] Richburg AG Kelly DP Davis-Kean PE Richburg A Depression, Anxiety, and Pubertal Timing: Current Research and Future Directions. University of Michigan Undergraduate Research Journal. 2021;15:39–53. 10.3998/umurj.1383

[12] Alshammari AS Piko BF Fitzpatrick KM Social support and adolescent mental health and well-being among Jordanian students. Int J Adolesc Youth. 2021;26:211–23. 10.1080/02673843.2021.1908375

[13] Maheshwari SK Chaturvedi R Gupta S Impact of family environment on mental well-being of adolescent girls: A cross-sectional survey. Indian Journal of Psychiatric Nursing. 2020;17:24. 10.4103/IOPN.IOPN_19_19

[14] Basu S Banerjee B Impact of environmental factors on mental health of children and adolescents: A systematic review. Child Youth Serv Rev. 2020;119:105515. 10.1016/j.childyouth.2020.105515

[15] Jahan N Went TR Sultan W Sapkota A Khurshid H Qureshi IA Untreated depression during pregnancy and its effect on pregnancy outcomes: a systematic review. Cureus. 2021;13:e17251. 10.7759/cureus.17251 34540477 PMC8448270

[16] Shriyan P Sudhir P van Schayck OCP Babu GR Association of high cortisol levels in pregnancy and altered fetal growth. Results from the MAASTHI, a prospective cohort study, Bengaluru. Lancet Reg Health Southeast Asia. 2023;14:100196. 10.1016/j.lansea.2023.100196 37461746 PMC7614758

[17] Su Q Zhang H Zhang Y Zhang H Ding D Zeng J Maternal stress in gestation: birth outcomes and stress-related hormone response of the neonates. Pediatr Neonatol. 2015;56:376–81."https://doi.org/10.1016/j.pedneo.2015.02.002" 10.1016/j.pedneo.2015.02.002 26363772

[18] Kågesten A Gibbs S Blum RW Moreau C Chandra-Mouli V Herbert A Understanding factors that shape gender attitudes in early adolescence globally: A mixed-methods systematic review. PLoS One. 2016;11:e0157805. 10.1371/journal.pone.0157805 27341206 PMC4920358

[19] United Nations Children’s Fund. Ending child marriage: Progress and prospects. New York, NY, USA: United Nations Children’s Fund; 2014.

[20] Datzberger S Le Mat ML Just add women and stir?: Education, gender and peacebuilding in Uganda. Int J Educ Dev. 2018;59:61–9. 10.1016/j.ijedudev.2017.09.006

[21] Rahman M Jamil K Nahar Q Chakraborty N Haider MM Khan S Factors that provide protection against intimate partner physical violence among married adolescents in Bangladesh. Front Public Health. 2023;11:1125056. 10.3389/fpubh.2023.1125056 37077187 PMC10106669

[22] Data for Impact. Bangladesh Adolescent Health and Wellbeing Survey 2019-20. 2021. Available: https://dataverse.unc.edu/dataset.xhtml;jses%20sionid=2041ade5046836520c1117daa52b?persistentId=doi%3A10.15139%2FS3%2FDVEI9A&version=&q=&fileTypeGroupFacet=%22Tabular+Data%22&fileAccess=&fileTag=&fileSortField=&fileSortOrder=. Accessed: 5 May 2025.

[23] Horwitz AV McLaughlin J White HR How the negative and positive aspects of partner relationships affect the mental health of young married people. J Health Soc Behav. 1998;39:124–36. 10.2307/2676395 9642903

[24] Qadir F Khalid A Haqqani S Medhin G The association of marital relationship and perceived social support with mental health of women in Pakistan. BMC Public Health. 2013;13:1150. 10.1186/1471-2458-13-1150 25226599 PMC3890521

[25] Pateraki E, Roussi P. Marital quality and well-being: The role of gender, marital duration, social support and cultural context. In: Efklides A, Moraitou D, editors. A positive psychology perspective on quality of life. Dordrecht, the Netherlands: Springer Dordrecht; 2012. p. 125–45.

[26] Dixit A Bhan N Benmarhnia T Reed E Kiene SM Silverman J The association between early in marriage fertility pressure from in-laws’ and family planning behaviors, among married adolescent girls in Bihar and Uttar Pradesh, India. Reprod Health. 2021;18:60. 10.1186/s12978-021-01116-9 33750403 PMC7941884

[27] Samandari G Sarker BK Grant C Huq NL Talukder A Mahfuz SN Understanding individual, family and community perspectives on delaying early birth among adolescent girls: findings from a formative evaluation in rural Bangladesh. BMC Womens Health. 2020;20:169–10. 10.1186/s12905-020-01044-z 32778096 PMC7419185

[28] Henry EG Lehnertz NB Alam A Ali NA Williams EK Rahman SM Sociocultural factors perpetuating the practices of early marriage and childbirth in Sylhet District, Bangladesh. Int Health. 2015;7:212–7. 10.1093/inthealth/ihu074 25294844

[29] Bennett HA Einarson A Taddio A Koren G Einarson TR Prevalence of depression during pregnancy: systematic review. Obstet Gynecol. 2004;103:698–709. 10.1097/01.AOG.0000116689.75396.5f 15051562

[30] Maslow AH. Motivation And Personality: Motivation And Personality: Unlocking Your Inner Drive and Understanding Human Behavior. New Delhi, India: Prabhat Prakashan; 2023.

[31] Andrews B Bodily shame as a mediator between abusive experiences and depression. J Abnorm Psychol. 1995;104:277. 10.1037/0021-843X.104.2.277 7790630

[32] Cunha M Matos M Faria D Zagalo S Shame memories and psychopathology in adolescence: The mediator effect of shame. Int J Psychol Psychol Ther. 2012;12:203–18.

[33] Graham S Victims of bullying in schools. Theory Pract. 2016;55:136–44. 10.1080/00405841.2016.1148988

[34] Rachman LY Widianti E Setyawati A Levels of depression among adolescent girls with early marriage. Journal of Maternity Care and Reproductive Health. 2019;2:223–233. 10.36780/jmcrh.v2i3.82

[35] Patel P Bhattacharyya K Singh M Jha RP Dhamnetiya D Shri N Depression among currently married ever pregnant adolescents in Uttar Pradesh and Bihar: Evidence from understanding the lives of adolescents and young adults (UDAYA) survey, India. Indian J Psychiatry. 2024;66:148–56. 10.4103/indianjpsychiatry.indianjpsychiatry_176_23 38523760 PMC10956582

[36] Nishat JF Shovo T-E-A Ahammed B Islam MA Rahman MM Hossain MT Mental health status of early married girls during the COVID-19 pandemic: A study in the southwestern region of Bangladesh. Front Psychiatry. 2023;13:1074208. 10.3389/fpsyt.2022.1074208 36683997 PMC9849885

[37] Khan JR Muurlink O Hu N Awan N Lingam R Pregnancy, spousal connectedness, and young married women’s mental health: an analysis of the Bangladesh adolescent health and wellbeing survey. Arch Womens Ment Health. 2023;26:235–44. 10.1007/s00737-023-01302-7 36930396

[38] United Nations. World Population Prospects 2024. Available: https://population.un.org/wpp/downloads?folder=Standard%20Projections&group=Population. Accessed: 28 June 2025.

[39] National Institute of Population Research and Training (NIPORT), ICF. Bangladesh Demographic and Health Survey 2022: Key Indicators Report. Dhaka, Bangladesh, Rockville, Maryland, USA: NIPORT, ICF; 2023. Available: https://dhsprogram.com/pubs/pdf/PR148/PR148.pdf. Accessed: 5 May 2025.

[40] Mallik CI Radwan RB Adolescent victims of cyberbullying in Bangladesh-prevalence and relationship with psychiatric disorders. Asian J Psychiatr. 2020;48:101893. 10.1016/j.ajp.2019.101893 31865200

[41] Nahar P Van Reeuwijk M Reis R Contextualising sexual harassment of adolescent girls in Bangladesh. Reprod Health Matters. 2013;21:78–86. 10.1016/S0968-8080(13)41696-8 23684190

[42] Naved RT Persson LÅ Factors associated with spousal physical violence against women in Bangladesh. Stud Fam Plann. 2005;36:289–300. 10.1111/j.1728-4465.2005.00071.x 16395946

[43] World Health Organization. Putting women first: Ethical and safety recommendations for research on domestic violence against women. Geneva, Switzerland: World Health Organization; 2001. Available: https://www.who.int/publications/i/item/WHO-FCH-GWH-01.1. Accessed: 5 May 2025.

[44] Spitzer RL, Williams JBW, Kroenke K. Patient Health Questionnaire (PHQ) Screeners. 2025. Available: https://www.phqscreeners.com/select-screener. Accessed: 5 May 2025.

[45] Johnson JG Harris ES Spitzer RL Williams JB The patient health questionnaire for adolescents: validation of an instrument for the assessment of mental disorders among adolescent primary care patients. J Adolesc Health. 2002;30:196–204. 10.1016/S1054-139X(01)00333-0 11869927

[46] Axelson O Fredriksson M Ekberg K Use of the prevalence ratio v the prevalence odds ratio as a measure of risk in cross sectional studies. Occup Environ Med. 1994;51:574. 10.1136/oem.51.8.574 7951785 PMC1128040

[47] Miettinen OS Cook EF Confounding: essence and detection. Am J Epidemiol. 1981;114:593–603. 10.1093/oxfordjournals.aje.a113225 7304589

[48] Barros AJ Hirakata VN Alternatives for logistic regression in cross-sectional studies: an empirical comparison of models that directly estimate the prevalence ratio. BMC Med Res Methodol. 2003;3:21. 10.1186/1471-2288-3-21 14567763 PMC521200

[49] Lee J Estimation of prevalence rate ratios from cross-sectional data: a reply. Int J Epidemiol. 1995;24:1066–7. 10.1093/ije/24.5.1066 8557441

[50] Campbell JC Health consequences of intimate partner violence. Lancet. 2002;359:1331–6. 10.1016/S0140-6736(02)08336-8 11965295

[51] Ellsberg M Jansen HA Heise L Watts CH Garcia-Moreno C Intimate partner violence and women’s physical and mental health in the WHO multi-country study on women’s health and domestic violence: an observational study. Lancet. 2008;371:1165–72. 10.1016/S0140-6736(08)60522-X 18395577

[52] Wisner KL Gelenberg AJ Leonard H Zarin D Frank E Pharmacologic treatment of depression during pregnancy. JAMA. 1999;282:1264–9. 10.1001/jama.282.13.1264 10517430

[53] Mahendran R Puthussery S Amalan M Prevalence of antenatal depression in South Asia: a systematic review and meta-analysis. J Epidemiol Community Health. 2019;73:768–77. 10.1136/jech-2018-211819 31010821

[54] Kaltiala-Heino R Fröjd S Marttunen M Sexual harassment and emotional and behavioural symptoms in adolescence: stronger associations among boys than girls. Soc Psychiatry Psychiatr Epidemiol. 2016;51:1193–201. 10.1007/s00127-016-1237-0 27229888

[55] Lichty LF Campbell R Targets and witnesses: Middle school students’ sexual harassment experiences. J Early Adolesc. 2012;32:414–30. 10.1177/0272431610396090

[56] Chiodo D Wolfe DA Crooks C Hughes R Jaffe P Impact of sexual harassment victimization by peers on subsequent adolescent victimization and adjustment: A longitudinal study. J Adolesc Health. 2009;45:246–52. 10.1016/j.jadohealth.2009.01.006 19699420

[57] You S Lee Y Kim E Physical, social, and cyberbullying: Relationships with adolescents’ psychosocial factors. Child Indic Res. 2016;9:805–23. 10.1007/s12187-015-9338-y

[58] Gámez-Guadix M Orue I Smith PK Calvete E Longitudinal and reciprocal relations of cyberbullying with depression, substance use, and problematic internet use among adolescents. J Adolesc Health. 2013;53:446–52. 10.1016/j.jadohealth.2013.03.030 23721758

[59] National Institute of Population Research and Training (NIPORT), ICF. Bangladesh Demographic and Health Survey 2017-18. Dhaka, Bangladesh, Rockville, Maryland, USA: NIPORT, ICF; 2020. Available: https://dhsprogram.com/pubs/pdf/FR344/FR344.pdf. Accessed: 5 May 2025.

[60] Khan S, Haider MM, Jamil K, Ahsan KZ, Angeles G. The double burden of malnutrition among Bangladeshi women: Rethinking the country’s maternal and child health programs and policies. Data for impact. 2022. Available: https://www.data4impactproject.org/publications/the-double-burden-of-malnutrition-among-bangladeshi-women-rethinking-the-countrys-maternal-and-child-health-programs-and-policies/. Accessed: 5 May 2025.

[61] Robertson E Grace S Wallington T Stewart DE Antenatal risk factors for postpartum depression: a synthesis of recent literature. Gen Hosp Psychiatry. 2004;26:289–95. 10.1016/j.genhosppsych.2004.02.006 15234824

[62] Grigoriadis S VonderPorten EH Mamisashvili L Tomlinson G Dennis CL Koren G The impact of maternal depression during pregnancy on perinatal outcomes: a systematic review and meta-analysis. J Clin Psychiatry. 2013;74:e321. 10.4088/JCP.12r07968 23656857

[63] Hossain AT Rahman MH Manna RM Akter E Islam SMH Hossain MA Enhancing Access to Mental Health Services for Antepartum and Postpartum Women Through Telemental Health Services at Wellbeing Centers in Selected Health Facilities in Bangladesh: Implementation Research. JMIR Pediatr Parent. 2025;8:e65912. 10.2196/65912 39753209 PMC11748442

[64] Shamim I. Child sexual abuse and exploitation online in Bangladesh: The challenges of the internet and law and legal developments. In: Shahidullah SM, editor. Crime, Criminal Justice, and the Evolving Science of Criminology in South Asia: India, Pakistan, and Bangladesh. London, UK: Palgrave Macmillan; 2017. p. 145–71.

[65] World Health Organization. Bangladesh WHO Special Initiative for Mental Health Situational Assessment. 2020. Available: https://www.who.int/docs/default-source/mental-health/special-initiative/who-special-initiative-country-report—bangladesh—2020.pdf?sfvrsn=c2122a0e_2. Accessed: 5 May 2025.

[66] Government of the People’s Republic of Bangladesh, Directorate General of Family Planning. National mental health strategic plan 2020-2030. Bangladesh: Government of the People’s Republic of Bangladesh, Directorate General of Family Planning; 2023. Available: https://dghs.gov.bd/site/notices/a315454c-eede-43da-b5a6-501bef5e1a1e/National-Mental-Health-Strategic-Plan-2020-2030. Accessed: 5 May 2025.

[67] Government of the People’s Republic of Bangladesh, Ministry of Health and Family Welfare. National Strategy for Adolescent Health 2017-2030. Dhaka, Bangladesh: Government of the People’s Republic of Bangladesh, Directorate General of Family Planning; 2016. Available: https://www.unicef.org/bangladesh/sites/unicef.org.bangladesh/files/2018-10/National-Strategy-for-Adolescent-Health-2017-2030.pdf. Accessed: 5 May 2025.

[68] Sidebottom AC Harrison PA Godecker A Kim H Validation of the Patient Health Questionnaire (PHQ)-9 for prenatal depression screening. Arch Womens Ment Health. 2012;15:367–74. 10.1007/s00737-012-0295-x 22983357

[69] Wang L Wu T Anderson JL Florence JE Prevalence and risk factors of maternal depression during the first three years of child rearing. J Womens Health (Larchmt). 2011;20:711–8. 10.1089/jwh.2010.2232 21426237

[70] Wang Y Wei R Chen Z Tang Y Liu L Qiao P The association between the number of pregnancies and depressive symptoms: A population-based study. J Affect Disord. 2024;350:411–9. 10.1016/j.jad.2024.01.161 38244784

